# Impacts of Sociodemographic Factors, Screening, and Organization of Health Services on Breast Cancer Mortality in Brazil: An Ecological Study of 20 Years

**DOI:** 10.1155/2023/6665725

**Published:** 2023-10-30

**Authors:** Thalita da Luz Costa, Diego Bessa Dantas, Fabiana de Campos Gomes, Cleuma Oliveira Soares, Janielly Reis Castelhano, Laryssa Corrêa Fonseca, Laura Maria Tomazi Neves, Eric Renato Lima Figueiredo, João Simão de Melo Neto

**Affiliations:** ^1^Institute of Health Sciences, Federal University of Pará (UFPA), Belém, PA, Brazil; ^2^Faculty of Medicine of São José do Rio Preto (FAMERP), São José do Rio Preto, São Paulo, Brazil

## Abstract

**Background:**

Breast cancer mortality is increasing in Brazil. This study examines the impact of sociodemographic factors, screening procedures, and primary healthcare (PHC) on breast cancer mortality.

**Methods:**

An ecological study analyzed secondary data of women diagnosed with breast cancer who died between 2000 and 2019. Sociodemographic factors, screening procedures, and PHC were examined in relation to breast cancer mortality. Statistical analyses included normality tests, Kruskal-Wallis and one-way ANOVA tests with post hoc comparisons, Pearson and Spearman correlation tests, age-period-cohort analysis, Kaplan-Meier analysis, and Cox regression analysis. Significance was set at *p* < 0.05.

**Results:**

Mortality rates were higher in the southeast (15.77) and south (15.97) regions compared to the north (5.07) (*p* < 0.0001). Survival rates were longer in the southeast (70.3 ± 0.05) and south (70.6 ± 0.09) than in the north (63.98 ± 0.053) (*p* ≤ 0.001). Mortality increased with age after 32 years (*p* ≤ 0.001). Brown and indigenous women had lower mortality and survival rates. Increased coverage of PHC, ultrasound, and biopsy did not reduce mortality. However, improved cytopathologic analysis led to a decrease in mortality.

**Conclusions:**

Sociodemographic factors, screening procedures, and PHC are specific predictors of breast cancer mortality in Brazil.

## 1. Introduction

Breast cancer stands as the leading cause of cancer-related deaths among women globally. Alarmingly, the majority of these fatalities, approximately 60%, are concentrated in developing nations. In these countries, the five-year survival rate is a mere 40%, a stark contrast to the 80% observed in developed nations [1]. While many developed countries have witnessed a decline in breast cancer mortality, Brazil has experienced a surge over the past three decades [2].

This disparity in mortality rates across Brazil can be attributed to a myriad of factors, including geographical location, demographic shifts, socioeconomic conditions, and cultural differences [2]. A comprehensive understanding of these determinants and awareness of risk factors, demographic characteristics, and the accessibility of health services are pivotal for effective breast cancer prevention and treatment [3, 4].

Diagnostic procedures for breast cancer encompass a triad: physical breast examination, imaging tests (namely mammography and ultrasound), and breast cytology [5]. However, the prohibitive costs of imaging tests can impede accurate diagnosis for economically disadvantaged patients [4]. Despite the Brazilian Unified Health System (Sistema Único de Saúde [SUS]) offering these tests at no charge [6], the nation grapples with infrastructural deficits, inadequate funding, and limited initiatives promoting cancer screening and treatment [1], all of which can exacerbate mortality rates. SUS, the world's sole free public health system, caters to over 190 million individuals [6]. It facilitates mammography, the gold standard for breast cancer detection and prevention [7], and ultrasound, a supplementary imaging test. When used in tandem, these tests enhance diagnostic accuracy [8], especially as ultrasounds can detect elusive invasive tumors and nodules in younger women or those with dense breast tissue [9].

However, a glaring disparity exists in the distribution of mammography devices across Brazil. In 2016, of the 2,113 registered mammographs, the southeast region boasted 39%, while the northern region had a mere 6% [10, 11]. This inequitable distribution prompts a pressing question: “Does the national disparity in resource allocation and utilization influence breast cancer mortality rates?”

Technological strides in imaging notwithstanding, breast image evaluation still faces limitations. Often, a conclusive diagnosis necessitates a biopsy [12]. The prevalent method is the percutaneous biopsy, executed using a thick needle. In cases where needle biopsy is unfeasible, surgical biopsy becomes the recourse [13].

It is imperative to recognize that early detection and timely treatment significantly mitigate the risk of breast cancer mortality. Conversely, delayed diagnosis escalates the risk, often due to hindered access to health services [7]. Thus, gauging the influence of health service organization on mortality rates is crucial for devising effective strategies.

Given Brazil's vast territorial expanse and regional nuances, there is an urgent call for research pinpointing the factors influencing breast cancer mortality. This study endeavors to scrutinize the impact of social, demographic, and screening factors, as well as primary care coverage, on breast cancer mortality rates in Brazil. We hypothesize that factors such as nonwhite ethnicity, advanced age, regions with diminished per capita income, inadequate screening procedures, and limited primary care coverage exacerbate breast cancer mortality in Brazil.

## 2. Materials and Methods

### 2.1. Study Design

This study employed an ecological, descriptive, and inferential design to fulfill its objectives.

### 2.2. Population and Study Area

The focus of this study encompassed secondary data concerning women who had received a diagnosis of malignant breast cancer and subsequently succumbed to the disease in Brazil. The designation of malignant breast cancer adhered to code C50 as per the 10th revision of the International Classification of Diseases (ICD-10) [14]. The study was conducted considering the official administrative and geopolitical divisions of Brazil, which categorizes the country into five macroregions: north, northeast, midwest, southeast, and south. These divisions are established based on historical, cultural, and economic dynamics and are officially recognized for administrative purposes, including health surveillance and policy implementation.

### 2.3. Eligibility Criteria

Inclusion criteria encompassed patients who had passed away within the period spanning from 2000 to 2019 and had been categorized under ICD-10 code C50, indicative of malignant breast neoplasms [14]. Excluded from the analysis were deaths that had been registered beyond the defined study timeframe, alongside individuals for whom vital variables were absent in the dataset.

### 2.4. Data Collection

Annual acquisition of data occurred through the Mortality Information System (SIM), a sector within the Department of Informatics of the Unified Health System (DATASUS) in Brazil [15]. Furthermore, data were sourced from the IBGE Automatic Recovery System (SIDRA) [16] and e-Gestor AB, a platform for information and primary care management [17]. The collection took place between the months of December 2020 and February 2021.

### 2.5. Database

The DATASUS platform, an initiative established by the Ministry of Health, serves as a health repository that furnishes accessible public health data for the purpose of health analysis and program formulation [15]. The Brazilian Institute of Geography and Statistics (IBGE) undertakes the noble mission of disseminating sociodemographic, economic, and geospatial information [16]. Complementarily, the e-Gestor AB functions as a comprehensive hub for centralizing entries and profiles of primary care systems (AB). This platform also acts as a hub for relevant information catering to state and municipal administrators. The dataset accessible via e-Gestor AB emanated from the National Registry of Health Establishments System (SCNES) and IBGE [17].

### 2.6. Variables Analyzed

**Figure 1:**
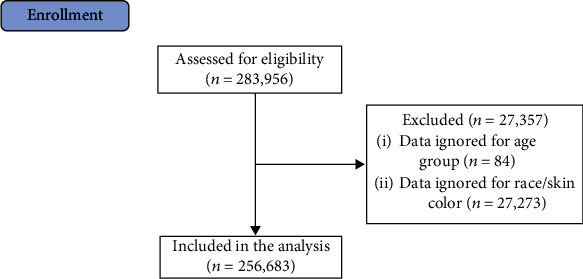
Flow diagram of sample eligibility.

The sociodemographic variables subjected to scrutiny encompassed geographic regions within Brazil, age groups, and race/skin color. The cumulative count of deaths pertinent to these variables amounted to 283,956. For the purpose of geographic region analysis (i.e., north, northeast, southeast, south, and midwest), no deaths were excluded from consideration. Pertaining to age groups (i.e., less than 19, 20–29, 30–39, 40–49, 50–59, 60–69, and 70 years old or more), the total number of deaths with unverified age stood at 84. Similarly, regarding race/skin color (i.e., brown, white, black, yellow, and indigenous), the sum of deaths omitted from the analysis equated to 27,273. A more comprehensible depiction of this is provided in the flowchart (Figure 1) elucidating the subjects of the study.

The breast cancer screening procedures employed by the Ministry of Health within the scope of this research encompassed the following components: anatomopathological examination of breast biopsies (included in the analysis: 335,474), bilateral breast ultrasounds (included in the analysis: 12,521,377), bilateral mammography for screening purposes (included in the analysis: 40,216,986), and cytopathological examination of breast tissues (included in the analysis: 269,343).

Population coverage estimates within the realm of primary care were derived from the proportion of the populace attended to by family health strategy and primary care teams, with equivalence and parameterization concerning population estimates.

Crude mortality rates, expressed per 100,000 female residents, were computed for each variable categorized by region, age group, and skin color/race. The standardization of these rates involved reference to mortality data. The dataset was sourced from the Mortality Information System (SIM) provided by DATASUS. Population data were gleaned from research conducted by the Brazilian Institute of Geography and Statistics (IBGE), leveraging demographic census records (2000 and 2010) and population estimates for years lacking census data.

### 2.7. Statistical Analysis

The obtained data underwent both descriptive and inferential analyses. Descriptive analysis encompassed the utilization of absolute and relative frequencies. Initially, the data underwent assessment via the Shapiro-Wilk normality test. Subsequently, the Kruskal-Wallis test, coupled with the post hoc Dunn test for nonparametric data or the one-way ANOVA test along with the post hoc Tukey test for parametric data, was applied to gauge heterogeneity across geographic regions, skin colors, and age groups. Relationships between variables were evaluated using the Pearson correlation (parametric) or Spearman correlation (nonparametric), with correlations categorized as low (*r* < 0.33), moderate (*r* between 0.34 and 0.66), or strong (*r* > 0.66). An age-period-cohort (APC) analysis was executed, incorporating the Wald test to discern significant disparities. The APC Web Tool, offered by the Biostatistics Branch of the National Cancer Institute, Bethesda, MD, USA [18], facilitated these estimations. Survival analysis was conducted, involving Kaplan-Meier and Cox regression analyses. Kaplan-Meier curve discrepancies were examined via the Log Rank (Mantel-Cox), Breslow (Generalized Wilcoxon), and Tarone-Ware tests. Statistical significance was predicated on a threshold of *p* < 0.05. The analytical tools employed encompassed GraphPad Prism 8 and SPSS Statistics.

### 2.8. Ethical Aspects

This study hinged on the analysis of secondary data procured through diverse channels, encompassing open access, disclosed usage, and unrestricted accessibility. Given that the secondary data was retrieved from open access information systems, the study was exempt from the requirement of approval by the National Commission of Ethics in Research of Brazil, in accordance with Resolution no. 510, dated April 7, 2016.

## 3. Results

### 3.1. Relationship between Sociodemographic Factors and Breast Cancer Mortality

**Figure 2:**
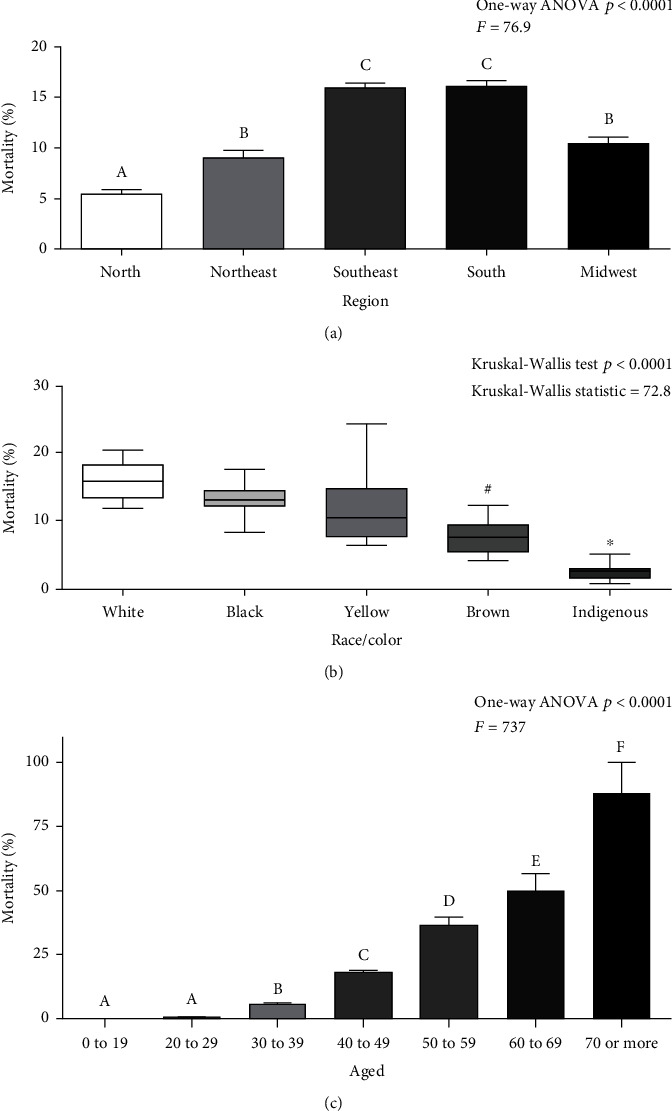
Mortality rates according to region, race/skin color, and age group between 2000 and 2019. Legend: (a) Mortality rates according to region; (b) mortality rates according to race/color; (c) mortality rates according age. (A, B, C, D, E, F) Statistical difference (*p* < 0.05, Tukey's test); statistical difference (*p* < 0.05, Dunn's test) in the comparison of brown versus white or black^#^ and indigenous versus white or black or yellow^∗^.

Figure 2 shows the graphs of breast cancer crude mortality rates according to region, race/color, and age between 2000 and 2019 in Brazil. Among the regions, the following crude mortality rates were observed: north (5.07), northeast (9.27), southeast (15.77), south (15.97), and midwest (10.28) (Figure 2(a)). The following types of race/skin color were observed: white (15.71), black (13.17), yellow (10.35), brown (7.49), and indigenous (2.55) (Figure 2(b)). Regarding the age group, the following are observed: from 0 to 19 (0.01), from 20 to 29 (0.56), from 30 to 39 (5.55), from 40 to 49 (18.26), from 50 to 59 (36.67), from 60 to 69 (49.81), and from 70 to 79 (85.61) (Figure 2(c)).

### 3.2. Age–Period–Cohort Analysis

**Figure 3:**
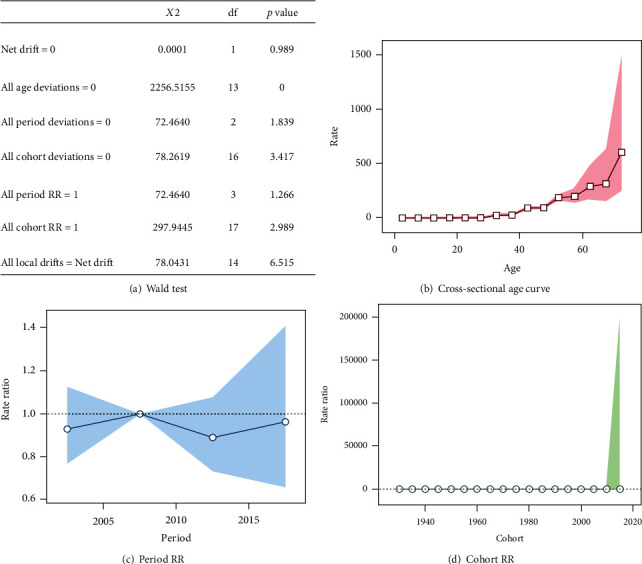
APC analysis utilizing a Wald test, a cross-sectional age curve, period RR, and cohort.

Figure 3 shows the results obtained from the APC analysis. It was observed that all age deviations presented *p* < 0.05, reinforcing that there is a greater tendency of mortality with increasing age from 32 years: 32 years (rate = 24.5629; 95% CI 13.1647 to 45.8297), 42 years (rate = 90.4655; 95% CI 70.4472 to 116.1723), 52 years (rate = 198.4248; 95% CI 161.3907 to 222.3285), 62 years (rate = 288.9658; 95% CI 170.4932 to 489.7628), and 72 years (rate = 602.3021; 95% CI 243.476 to 1489.953).

### 3.3. Association Analysis

Table 1 describes the results of the correlation between the mortality rate (2008 to 2019) and coverage of primary care, coverage of the family health strategy, and screening procedures performed for the diagnosis of breast cancer. Regarding health coverage, the primary care and family health strategy variables showed a strong positive correlation with mortality in all regions of Brazil (Table 1). The mammography showed no correlation with mortality in any region. The bilateral ultrasound examination of the breast showed a strong positive correlation with the southeast, south, and midwest regions. The anatomopathological examination of the breast (biopsy) showed a strong positive correlation with the mortality rate in almost all regions, except in the northern region. The cytopathological examination of the breast showed a strong positive correlation with the north region and a negative correlation with the southeast, south, and midwest regions.

### 3.4. Survival Analysis

The estimation of mean survival time (years) according to race was distributed in yellow (72.98 ± 0.46), white (71.43 ± 0.5), black (67.69 ± 0.15), indigenous (67.09 ± 1.40), and brown (65.64 ± 0.78). In relation to the regions, the estimation of mean survival time (years) was distributed in the south (70.60 ± 0.09), southeast (70.30 ± 0.05), northeast (68.11 ± 0.09), midwest (66.47 ± 0.16), and north (63.99 ± 0.20).

**Figure 4:**
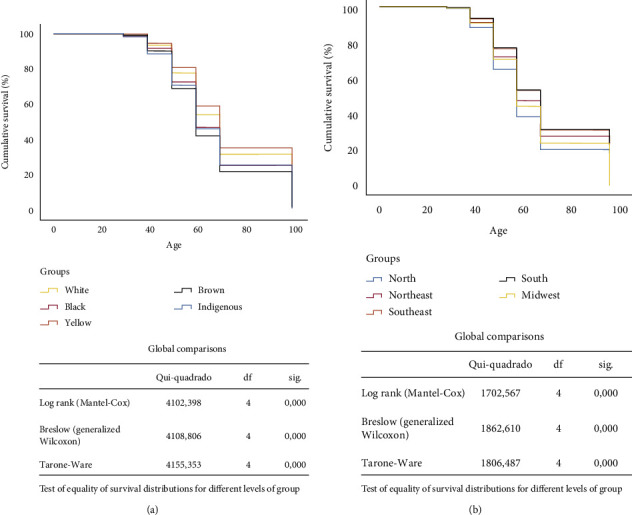
Survival in accordance with geographic regions and race groups in Brazil, between 1996 to 2019. Legend: (a) survival in accordance with race groups; (b) survival in accordance with geographic regions.

In Figure 4 and Table 2, we observe survival according to geographic regions and racial groups in Brazil, between 2000 and 2019, using a Cox regression. The hazard ratios and their respective confidence intervals (HR, 95%) are observed in Table 2. Regarding race/skin color, it is possible to state that brown women have a lower survival rate, while yellow and white women have a higher survival rate (Figure 4(a)). Regarding the geographic regions, it can be seen that the north region had a lower survival rate, while the south and southeast regions had a higher survival rate (Figure 4(b)).

## 4. Discussion

Within the Brazilian context, a disconcerting upswing in the mortality rate spanning the last three decades has come to the forefront [2]. In the quest for comprehensive insights, inquiries necessitate scrutinizing the sway exerted by social and demographic variables, screening methodologies, and the extent of primary care encompassing breast cancer mortality in Brazil. The present study casts a revealing light by deciphering that within this intricate tapestry, sociodemographic and clinical factors manifest distinctive attributes that furnish prognostic cues pertaining to breast cancer mortality within Brazil.

In the context of demographic factors, it emerges that the mortality rate holds a higher trajectory within the southeast and south regions of Brazil; paradoxically, these regions also exhibit an extended survival span for affected women. The observed trends in this study potentially arise from a significant migration flow of individuals seeking healthcare services, a phenomenon markedly pronounced in the southeast region, with São Paulo being a prominent recipient of this influx. This migration is largely driven by the search for better healthcare opportunities available in the region, which consequently impacts the breast cancer mortality rates documented in the study. Noteworthy is the temporal shift observed: while the year 2004 witnessed substantial transfers from other states in both the midwest and the southeast, by 2014, the focal point had evolved to be predominantly confined to the southeast alone. This nuanced transition bespeaks an augmentation in hospitalization practices across diverse regions, offering insight into the amplified mortality observed within the southeast region during this study [19].

Furthermore, it becomes evident that the southeast region contends with a notable paucity of mammography devices, instruments pivotal for the early identification of breast cancer [20]. Notably, the interval from 1998 to 2012 casts the south region as a trailblazer in terms of cancer incidence and mortality across Brazil, attaining global benchmarks for cancer-associated fatalities. Conversely, spanning the period from 1980 to 2010, an upward trajectory in breast cancer-related deaths within Brazil is juxtaposed against a decline in mortality rates within state capitals. However, municipalities witnessed a contrary surge in mortality. The genesis of this disparity can be attributed to the formidable challenges faced by rural populations in accessing healthcare networks, further compounded by the evolving regulations within the Brazilian Unified Health System (SUS), which diligently curbs the chaotic migration of patients to treatment hubs situated within capitals and more advanced states [21, 22].

Furthermore, it comes to light that the north region, while exhibiting lower mortality, conversely presents a diminished survival rate. The research conducted by Carvalho et al. [23] yields pertinent insights, delineating the predominant breast cancer subtypes across distinct Brazilian regions. In particular, the north region grapples with a preponderance of the most severe breast cancer variants, enriched with HER2 and characterized as triple-negative. In stark contrast, luminal tumors hold sway within the south and southeast regions, offering a more favorable prognosis [23].

The demographic tapestry unveils a telling narrative. The north region, predominantly comprised of individuals of indigenous and black ethnicities, stands juxtaposed to the south and southeast regions, wherein the demographic fabric leans predominantly towards the white ethnic demographic [23]. A comprehensive study encompassing 447 women aligns with these patterns, substantiating that triple-negative breast cancer prevalence is notably pronounced among nonwhite individuals with lower educational attainments. Strikingly, these women grappling with such a diagnosis face a truncated 5-year survival rate [24]. Importantly, the outcomes of the current study converge with these established findings, collectively weaving a coherent fabric that underscores the intricate interplay between demographic attributes, distinct breast cancer subtypes, and their consequential impact on survival trajectories.

Within the scope of this study, a noteworthy revelation emerges wherein women of brown ethnicity exhibit diminished mortality rates alongside reduced survival prospects within the country. Intriguingly, existing literature corroborates this narrative, reporting the lowest mortality rates among brown women [25]. However, a layered panorama unveils itself upon closer examination. While the brown population demonstrates lower mortality, their attenuated survival rates can potentially be traced back to multifaceted factors. These include socioeconomic dimensions, particularly the impact of low income, reliance on the public health sector, and a propensity for more advanced stages of cancer. This intricate interplay between demographic attributes and healthcare nuances mirrors an established rationale that has previously been associated with black women [26]. Notably, the discourse surrounding brown individuals necessitates a nuanced perspective, as they are emblematic of a composite blend of racial lineages. While the underlying causes of these disparities remain multifaceted and complex, warranting a comprehensive and concerted effort to unravel, it is hypothesized that the lower mortality rates among indigenous women might be partly attributed to a lesser exposure to HPV pathogens, a factor that has been implicated in breast cancer pathology in various studies [27]. Conversely, the higher mortality rates in white women could potentially be influenced by genetic predispositions, among other factors, thus necessitating a deeper exploration into the myriad of sociobiological factors at play to foster improved breast cancer outcomes for all women. On the other hand, higher mortality rates in white women may potentially be influenced by genetic predispositions, among other factors, thus necessitating a deeper exploration of the myriad of sociobiological factors at play to promote better breast cancer outcomes for all women, which unfortunately is not possible based on the design of an ecological study.

The higher survival rates in the south and southeast regions, despite notable mortality rates, might be due to superior healthcare infrastructure and a population with better socioeconomic status, which ensures greater access to quality healthcare. These factors can significantly enhance survival rates, pointing to a necessity for improved healthcare facilities in other regions to bridge this gap [24]. When we turn our attention to racial disparities, it becomes evident that a range of factors including genetic predispositions and differential access to healthcare might be influencing the lower survival rates among brown women compared to yellow and white women. Addressing this requires a deep understanding of the multifaceted influences at play and a concerted effort to ensure equitable health outcomes for all racial groups [21].

It was also found in this study that mortality increases with age, with the rate escalating after the age of 32, and reaching its pinnacle beyond 70 years. The correlation between advanced age and heightened mortality has been elucidated by several studies [28–31]. Notably, women in nonreproductive phases exhibited elevated mortality rates [29], thereby warranting contemplation of hormonal fluctuations. Breast cancer, indeed, reigns as the primary cause of cancer-related fatalities among women aged under 45 years. APC analysis illuminated a discernible trend towards heightened mortality beyond the age of 32 within the Brazilian populace. The escalating incidence of breast cancer mortality within young Brazilian women, in consonance with global trends, has conspicuously unfolded over the past two decades [32]. This breed of cancer presents as exceptionally heterogeneous, characterized by intricate and potentially aggressive biological attributes. Nevertheless, the paradigm of management strategies, recommendations, and alternatives remains disentangled from age considerations. As such, the intricate biology underpinning this class of cancer persists as an arena steeped in uncertainty and uncharted exploration [31].

The population's awareness regarding signs and symptoms, the professional competence for diagnosis, and the healthcare system's capacity to deliver diagnostic and treatment services are inherently intertwined with the quality of care dispensed within primary care settings. Consequently, certain public policies enacted within the sphere of primary care can wield a potent impact on enabling early diagnosis [33]. Moreover, the World Health Organization and the Pan American Health Organization both underscore the potential of primary care as a platform to accentuate prevention and health promotion across the populace. Furthermore, primary care presents an avenue to cultivate healthful behaviors and lifestyles [34, 35].

An illustrative instance lies in the utilization of breast self-examination and clinical breast examination as diagnostic and screening modalities, especially in cases where mammographic diagnosis proves unfeasible. Nonetheless, a conspicuous void persists as many women remain unacquainted with these methodologies, thereby unveiling an inherent deficiency in propelling health education initiatives within the primary care domain. Notwithstanding the pronounced significance of prevention, this study unearths a paradox: even in the face of amplified coverage within the primary care and family health strategy realms, the mortality rate sustains an elevated trajectory [36].

This outcome resonates with findings from the study conducted by Figueiredo et al. [33]. In that investigation, an exploration of the interplay between primary healthcare (PHC) coverage, the family health team (FHS), and community health agents (CHA) was undertaken to discern their connection with breast cancer mortality across Brazilian municipalities. The conclusions drawn highlighted a consistent association between all primary care metrics and breast cancer mortality. The rationale underlying this congruence could potentially be elucidated through the incremental augmentation of primary care indicators' coverage within Brazil, where the tangible effects necessitate a temporal trajectory for manifestation. A salient facet to consider is that a substantial proportion of diagnoses materializes within the precincts of primary care. Thus, the lateness of a diagnosis can inadvertently precipitate a surge in the mortality rate, regardless of the extent of primary health service coverage [33]. Nevertheless, the optimization of specialized services tailored to this cohort remains pivotal in effectively mitigating mortality rates.

Screening mammography surfaces as a potential instrument poised to mitigate mortality rates. Paradoxically, within the realm of the present study, the correlation between mammography and mortality remained conspicuously elusive across all regions subjected to scrutiny. A thought-provoking parallel emerges from the explorations undertaken by Miller and collaborators, who embarked upon an intricate comparative analysis between mortality rates and the efficacy of screening mammography. Remarkably, their conclusions resonated in harmony with the findings of this current investigation, collectively hinting that the yearly practice of mammography does not inherently translate into a discernible reduction in breast cancer-related mortality [37]. In tandem, an all-encompassing systematic review of randomized clinical trials buttressed this narrative, disclosing an overarching theme of ambiguity concerning the efficacy of screening interventions in curbing mortality rates. While the universality of these observations might not uniformly span global contexts, the crux of this discourse reverberates insistently: an imperative calls for robust inquiries that delve into the intrinsic indispensability of screening mammography [38].

Ultrasonography (US) assumes the mantle of a supplementary diagnostic modality, contingent upon the expertise of the operator, the caliber of the examination, and the currency of the equipment employed [39]. Notwithstanding its diagnostic prowess, US does not clinch endorsement as a screening methodology [40]. Within the contours of this investigation, an intricate panorama unveils itself, as the southeast, south, and midwest regions collectively divulge a distinctive trend: an incremental upswing in the frequency of US procedures correlates with a parallel escalation in mortality rates. This stark juxtaposition underscores that mortality rates manifest as decoupled from the sheer volume of procedures executed, reinforcing their independent dynamics [39].

Breast lesions engender a requisite for supplementary diagnostic exploration. In this intricate milieu, the tenets of biopsy and the subsequent pathology diagnosis converge, wielding the prowess to illuminate tumor-staging nuances [12]. Concomitantly, the efficacy of breast cytology unfolds as a potent diagnostic ally, extending its reach towards both neoplastic and nonneoplastic breast lesions [41]. Furthermore, the present study unfurls a revelatory spectacle: an ascending trajectory in the tally of breast cytopathological examinations administered corresponds with a commensurate attenuation in the mortality rate. This intriguing correlation is perhaps underpinned by the pivotal role that cytopathological examinations play in shaping prognostic paradigms and treatment delineations [41]. Curiously, the discernible surge in biopsy numbers, in contrast, remains unattended by a mirrored reduction in mortality rates. This nuanced phenomenon points towards the indispensable import of diligent tissue processing and meticulous postcollection analysis, which necessitate prompt execution. This insight underscores the criticality of expediting these integral steps in the diagnostic trajectory.

It is important to note that Brazil is a country with specific geographic, social, and political characteristics, and therefore, caution should be exercised in directly generalizing the results of this study to other populations. According to a study published by the International Agency for Research on Cancer (IARC), there will be approximately 2.3 million new cases of breast cancer and approximately 685,000 deaths from this disease worldwide in 2020, with large geographical variations observed between countries and regions of the world [42]. Breast cancer incidence rates are higher in countries that have undergone economic transition, but countries in transition have a disproportionate share of breast cancer deaths [42].

In this context, Brazil finds itself enmeshed in an intricate tapestry of internal disparities with regard to breast cancer mortality metrics. The influence exerted by socioeconomic factors upon breast cancer mortality outcomes resonates harmoniously with the findings gleaned from parallel Brazilian investigations, thereby coalescing into a collective narrative that strives to unravel shared patterns and incongruities [43, 44].

If we extend the geographical scale of analysis to Latin America, a study with projections to the end of the 2020s points out that Argentina, Uruguay, and Venezuela have the highest mortality rates, while Guatemala, El Salvador, and Nicaragua have the highest increases [45]. By considering these international studies alongside the Brazilian study, researchers can gain a more comprehensive understanding of breast cancer mortality trends, risk factors, and the effectiveness of interventions in different contexts.

Thus, we believe that among the strengths of this study are its potential for using public health data as a surveillance strategy for breast cancer mortality, providing subsidies for the application of other epidemiological studies with different types of design, as well as the use of public health data as a surveillance strategy for breast cancer mortality. It also helps to assess the consistency of socioeconomic inequality and access to health resources and services as a determinant of breast cancer mortality in different regions. This study can contribute to health services in Brazil by providing valuable information on breast cancer mortality and survival rates in different regions of the country. The results show that despite the increase in primary healthcare coverage, ultrasound, and biopsy procedures, there was no reduction in mortality. However, the increase in the performance of cytopathological analysis led to a reduction in the mortality rate. This suggests that improving the quality of healthcare can have a significant impact on reducing breast cancer mortality. An article published in the Revista Brasileira de Epidemiologia discusses the factors associated with poor access to health services by the Brazilian population and concludes that access to health services is still poor for a significant portion of the Brazilian population, especially the most vulnerable [46].

We underscore several limitations inherent to this study, premised upon its ecological design, thereby relying on secondary data. Such an approach invariably introduces constraints rooted in the integrity of data compilation. Within this context, certain data points remain shrouded in obscurity or elude reporting, exemplified by the conspicuous absence of information pertaining to social, demographic, and economic variables—this constitutes a pronounced drawback afflicting our research [47, 48]. Furthermore, it is imperative to acknowledge the emergence of biases or ecological fallacies stemming from the potentiality of engendering causal inferences with respect to individuals, predicated upon observations amassed from collective groupings. This phenomenon arises from the intricate interplay of several factors, including the heterogeneous dissemination of exposure to the subject of scrutiny and assorted confounding variables traversing these very groupings.

## 5. Conclusions

Mortality is higher in the southeast and south regions of Brazil; however, these women have a longer survival rate. The north has a lower mortality rate, but a lower survival rate. In addition, mortality increases with age, with the rate increasing after the age of 32. Regarding race/color, brown women had lower mortality and survival rates in the country. However, it was observed that even with the increase in the coverage of primary healthcare, ultrasound, and the number of biopsy procedures, there was no reduction in the mortality rate. However, an increase in the performance of cytopathology analysis led to a reduction in the mortality rate. Brazil is a country with unique geographic, social, and political characteristics. Therefore, caution should be exercised in directly generalizing the results of this study to other populations.

## Figures and Tables

**Table 1:** Correlation between mortality rate with screening, primary healthcare coverage, and procedures of diagnosis.

	North	Northeast	Southeast	South	Midwest
Primary care coverage	*p* ≤ 0.001^∗^	*p* ≤ 0.001^∗^	*p* ≤ 0.001^∗^	*p* ≤ 0.001^∗^	*p* ≤ 0.001^∗^
	S = 0.882	S = 0.909	P = 0.877	P = 0.913	P = 0.896
	95%CI = 0.5853 to 0.9703	95%CI = 0.6689 to 0.9774	95%CI = 0.5843 to 0.9677	95%CI = 0.6909 to 0.9774	95%CI = 0.6410 to 0.9730

Family health strategy coverage	*p* ≤ 0.001^∗^	*p* ≤ 0.001^∗^	*p* ≤ 0.001^∗^	*p* ≤ 0.001^∗^	*p* ≤ 0.001^∗^
	P = 0.959	P = 0.904	P = 0.907	S = 0.945	P = 0.975
	95%CI = 0.8451 to 0.9896	95%CI = 0.6642 to 0.9751	95%CI = 0.6730 to 0.9758	95%CI = 0.7908 to 0.9866	95%CI = 0.9019 to 0.9936

Bilateral mammography for screening	*p* = 0.077	*p* = 0.088	*p* = 0.107	*p* = 0.694	*p* = 0.464
	P = 0.553	S = 0.545	S = 0.145	S = −0.136	P = 0.247
	95%CI = −0.06962 to 0.8658	95%CI = −0.1012 to 0.8681	95%CI = −0.5131 to 0.6962	95%CI = −0.6914 to 0.5199	95%CI = −0.4141 to 0.7377

Bilateral breast ultrasound	*p* = 0.054	*p* = 0.114	*p* ≤ 0.001^∗^	*p* ≤ 0.001^∗^	*p* = 0.002^∗^
	P = 0.594	P = 0.504	P = 0.961	P = 0.961	P = 0.827
	95%CI = −0.008546 to 0.8804	95%CI = −0.1368 to 0.8478	95%CI = 0.8526 to 0.9901	95%CI = 0.8529 to 0.9901	95%CI = 0.4498 to 0.9536

Anatomopathological examination of the breast—biopsy	*p* = 0.129	*p* = 0.023^∗^	*p* = 0.0014^∗^	*p* < 0.0001^∗^	*p* = 0.003^∗^
	P = 0.487	P = 0.673	P = 0.835	S = 0.9406	P = 0.795
	95%CI = −0.1597 to 0.8411	95%CI = 0.1228 to 0.9068	95%CI = 0.4703 to 0.9559	95%CI = 0.7740 to 0.9854	95%CI = 0.3724 to 0.9444

Cytopathological examination of the breast	*p* ≤ 0.001^∗^	*p* = 0.487	*p* = 0.010^∗^	*p* = 0.005^∗^	*p* = 0.007^∗^
	P = 0.864	P = 0.235	P = −0.733	P = −0.781	P = −0.756
	95%CI = 0.5484 to 0.9642	95%CI = −0.4248 to 0.7317	95%CI = −0.9259 to -0.2381	95%CI = −0.9402 to -0.3400	95%CI = −0.9328 to -0.2858

Statistical difference (*p* < 0.05). P: Pearson's *r*; S: Spearman's *r*. ^∗^High correlation: P or S > 0.66. Moderate correlation: P or S 0.34 to 0.66. Low correlation: P or S < 0.33.

**Table 2:** Survival in accordance with geographic regions and race groups in Brazil, between 1996 and 2019.

	*p*	HR	95% CI
Race			
White and black	≤0.001	1.138	1.121 to 1.155
White and yellow	0.028	0.952	0.911 to 0.995
White and brown	≤0.001	1.229	1.218 to 1.240
White and indigenous	0.032	1.154	1.012 to 1.315
Black and yellow	≤0.001	0.845	0.807 to 0.885
Black and brown	≤0.001	1.076	1.059 to 1.093
Brown and yellow	≤0.001	0.785	0.751 to 0.821
Indigenous and black	0.835	0.986	0.865 to 1.125
Indigenous and yellow	0.006	0.825	0.719 to 0.947
Indigenous and brown	0.346	1.065	0.934 to 1.214
Region			
North and northeast	≤0.001	1.162	1.137 to 1.188
North and southeast	≤0.001	1.260	1.234 to 1.287
North and south	≤0.001	1.274	1.246 to 1.302
North and midwest	≤0.001	1.101	1.074 to 1.129
Northeast and southeast	≤0.001	1.077	1.067 to 1.088
Northeast and south	≤0.001	1.088	1.075 to 1.101
Northeast and midwest	≤0.001	1.063	1.045 to 1.082
Midwest and southeast	≤0.001	1.147	1.129 to 1.166
Midwest and south	≤0.001	1.157	1.137 to 1.178
South and southeast	0.063	1.010	0.999 to 1.020

## Data Availability

The data that support the findings of this study are available from DATASUS (http://www2.datasus.gov.br/DATASUS/index.php) and SIDRA IBGE (https://sidra.ibge.gov.br/home/ipca15/brasil).
